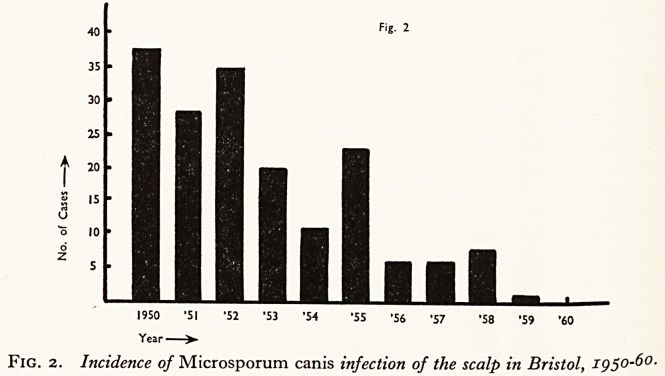# Fungus Infections of the Skin in Bristol and the West Country

**Published:** 1961-10

**Authors:** Mary P. English

**Affiliations:** Medical Mycologist, Bristol Royal Hospital


### FUNGUS INFECTIONS OF THE SKIN IN BRISTOL AND THE WEST COUNTRY

BY

MARY P. ENGLISH, M.SC.

*Medical Mycologist, Bristol Royal Hospital*

In 1954 a diagnostic service for fungal infections in man was set up in Bristol. It is available to any doctor, but most of the specimens submitted so far have been from dermatologists, and therefore the following account of the activities of the service in the last 6½ years is confined to fungal infections of the skin.

The object of the service is to provide facilities for the diagnosis of fungal infections of all types, and it can, therefore, be of use to E.N.T. surgeons, chest physicians, obstetricians, general practitioners, and others, as well as to dermatologists. The suggestions for the taking and despatch of specimens given in the appendix may be of interest to those who have not used the service before.

The correct diagnosis of fungal infections of the skin is of importance both for epidemiological and therapeutic reasons. Epidemiologically, if the species of fungus causing the infection is known, it may be possible to trace its origin and so to take suitable precautions against further spread: this is especially useful in detecting infections of animal origin and in tracing the source of cross-infection in tinea pedis. Therapeutically, the advent of griseofulvin, the new orally administered antifungal antibiotic, which is specific for ringworm infections and useless in any other condition, has rendered the correct diagnosis of these infections increasingly important. Griseofulvin therapy is prolonged and expensive, and as it is often difficult to diagnose fungal infection on clinical grounds alone, especially those of the feet and nails, laboratory proof of the presence of fungi before embarking on griseofulvin therapy is very desirable.

### THE MATERIAL EXAMINED

The origin of the patients is shown in the Tables. The various sources in Bristol have been using the service since its inception; specimens were first received from Cornwall and Weston-super-Mare in 1956, and from North Devon in 1958. Only the figures from Bristol General Hospital give a complete picture of the incidence of

TABLE I

Sites from which fungi were isolated in 810 infected patients (excluding cases of moniliasis)

| Site | SOURCE: Bristol General Hospital, 1954–1960 | Other Bristol sources, 1954–1960 | West Cornwall, 1956–1960 | North Devon 1958–1960 | Weston-super-Mare, 1954–1960 | Private Patients, 1954–1960 | Total |
|---|---|---|---|---|---|---|---|
| Scalp | 77 | 7(+17)* | 34 | 3 | 1 | 4 | 126(+17)* |
| Face | 48 | | 19 | 4 | | 1 | 72 |
| Limbs and Trunk | 122 | 14 | 53 | 6 | 1 | 12 | 208 |
| Hands | 36 | 3 | 15 | 4 | 1 | 16 | 75 |
| Finger-nails | 34 | 6 | 4 | 8 | 4 | 17 | 73 |
| Feet | 138 | 39 | 69 | 21 | 13 | 40 | 320 |
| Toe-nails | 51 | 11 | 32 | 18 | 5 | 4 | 131 |
| Groin and Perianal | 21 | 7 | 13 | 5 | 2 | 8 | 56 |
| Total .. | 527 | 87(+17)* | 239 | 69 | 27 | 112 | 1061(+17)* |

* An outbreak of scalp ringworm in a children's residential home.

mycoses in a dermatological clinic; those from all other sources represent the personal selection of cases by the dermatologists concerned.

Material from the skin or from skin appendages was received from 2,987 patients, and yielded a mycologically positive result in 999 cases (33·4 per cent). Two or more specimens were often received from the same patient.

In 810 of the patients the fungi isolated were those of the dermatophyte group. Table I sets out, for each source of specimens in this group, the number of occasions on which fungi were found in various parts of the body—often on more than one site in the same patient. Table II shows, under the same headings, the number of patients from whom the various dermatophytes were isolated.

TABLE II

Number of patients from whom each fungus was isolated (excluding cases of moniliasis)

| FUNGUS | SOURCE | | | | | | |
|---|---|---|---|---|---|---|---|
| | Bristol General Hospital | Other Bristol sources | West Cornwall | North Devon | Weston-super-Mare | Private Patients | Total |
| *Microsporum audouini* . . | 10 | 2(+17)* | 1 | 0 | 0 | 0 | 13(+17)* |
| *M. canis* . . | 66 | 4 | 17 | 3 | 1 | 3 | 94 |
| *Trichophyton mentagrophytes* | 19 | 3 | 16 | 0 | 0 | 2 | 40 |
| ,, ,, (*interdigitale* type) . . | 51 | 17 | 26 | 3 | 7 | 19 | 123 |
| *T. persicolor* . . | 2 | 0 | 0 | 0 | 0 | 0 | 2 |
| *T. rubrum* . . | 93 | 15 | 29 | 15 | 6 | 32 | 190 |
| *T. tonsurans* . . | 11 | 0 | 1 | 0 | 0 | 0 | 12 |
| *T. verrucosum* | 84 | 4 | 47 | 3 | 1 | 4 | 143 |
| *T. violaceum* . . (exotic origin) | 6 | 0 | 0 | 0 | 0 | 0 | 6 |
| *T. ferrugineum* (exotic origin) | 0 | 1 | 0 | 0 | 0 | 0 | 1 |
| *T. soudanense* . . (exotic origin) | 1 | 0 | 0 | 1 | 0 | 1 | 3 |
| *Epidermophyton floccosum* . . | 29 | 3 | 15 | 1 | 1 | 2 | 51 |
| *Scopulariopsis brevicaulis* . . (toe-nails) | 1 | 1 | 6 | 0 | 0 | 0 | 8 |
| *Aspergillus spp.* (toe- and finger-nails) | 7 | 2 | 3 | 1 | 1 | 0 | 14 |
| *Malassezia furfur* (pityriasis versicolor) | 20 | 4 | 16 | 0 | 0 | 0 | 40 |
| Micropscopic examination +ve, cultures failed . . | 34 | 10 | 21 | 9 | 3 | 6 | 83 |
| Total . . | 434 | 67(+17) | 198 | 36 | 20 | 69 | 823(+17)* |

* In 13 cases, 2 fungi were isolated from one patient; these isolations have been recorded separately in the Table, hence the discrepancy between the grand totals in this Table and Table I. The double infections consisted of

5 cases *E. floccosum* + *T. rubrum*.
4 cases *E. floccosum* + *T. mentagrophytes* (*interdigitale* type).
2 cases *T. rubrum* + *T. mentagrophytes* (*interdigitale* type).
1 case *M. canis* + *T. mentagrophytes* (*interdigitale* type).
1 case *T. verrucosum* + *T. persicolor*.

* An outbreak of scalp ringworm in a children's residential home.

In 189 patients, species of *Candida* were isolated, and these are described more fully below.

Though every fungus isolated has been recorded in the Tables, only those of common occurrence will be discussed in detail, under the heading of the diseases to which they give rise.

### TINEA CAPITIS

Fungi were isolated from specimens from the scalp in 126 patients. Of these fungi, 81 (65 per cent) were of the "small-spore" type, fluorescent under Wood's Lamp, and were of one or other species of *Microsporum*. Thirty-two (26 per cent) isolations were of *Trichophyton verrucosum*, the agent of cattle ringworm, and will be discussed later. The remainder were "large spore", non-fluorescent infections due to fungi of sporadic occurrence only. *M. audouini* is strictly a parasite of humans, thriving in institutions and in conditions of overcrowding and poverty. Only 13 cases have been seen in the general public in the 6½ years of the service, though there was an outbreak in a home for mentally deficient children, involving 17 patients, during this period. *M. canis*, which was the cause of 68 scalp infections, is primarily a parasite of cats and dogs, and is probably more often contracted direct from the infected animal than passed from child to child (Marples, 1959). In Bristol, as is shown in Fig. 1, there has been a dramatic fall in *M. audouni* infections of the scalp since the war, thanks to the cessation of "shelter" life, the return of "evacuated" children, the vastly improved living conditions, and a campaign of eradication centred on the schools and the General Hospital. The disease is now very rare in the city. Figure 2 shows the incidence of *M. canis* infection of the scalp in Bristol over the same period. While this fungus has never occurred as commonly as *M. audouini*, it has been less easy to stamp it out completely, because of the difficulty of controlling the animal vectors (La Touche, 1955). For instance, the comparatively large number of cases in 1955 mostly occurred in one street and were traced to two infected dogs.

### TINEA PEDIS

With the near-eradication of tinea capitis, tinea pedis is now the most common fungous disease of urban areas. Three hundred and twenty cases have been diagnosed in the whole district, and of all the fungous infections seen at the Bristol General Hospital, 32 per cent were tinea pedis.

By no means all cases of maceration and scaling of the toe-webs can be attributed to fungal infection, and accurate diagnosis based on clinical examination alone is extremely difficult. In a large proportion of cases so diagnosed, laboratory examination fails to disclose any evidence of fungi on repeated examination. This can be illustrated by figures taken from a survey of 4,794 day-school children in Bristol, where it was found that 34·7 per cent had lesions which, though rarely causing symptoms, could clinically have been those of tinea pedis; but fungi were in fact found in only 11·6 per cent of these lesions, i.e. in 3·6 per cent of all children (English and Gibson, 1959). The difficulty arises also in dermatological clinics. For instance, in 1958 (a typical year) 197 scrapings from feet from all over the area were examined in the diagnostic service, but in only 65 (33 per cent) were fungi present. Admittedly, many of these specimens were accompanied by a request to "exclude fungous infection", but clinically the element of doubt was there. Many other workers report comparable findings.

Tinea pedis can be caused by three fungi: the *interdigitale* type of *Trichophyton mentagrophytes*, *T. rubrum*, and *Epidermophyton floccosum*. In surveys of the population at large the first of the three is found to be very much more common than the other two (85 per cent of isolations from the school children mentioned above). In skin

FIG. 1. *Incidence of* Microsporum audouini *infection of the scalp in Bristol, 1950-60 (excluding 17 cases in a residential home).*

clinics, however, the *interdigitale* type of *T. mentagrophytes* and *T. rubrum* were isolated from feet in almost equal numbers. This inconsistency can be attributed to the chronic and disfiguring nature of most *T. rubrum* infections, which causes a high proportion of patients to seek specialist advice, while the great majority of *T. mentagrophytes* infections are treated by the family doctor or by the patient himself, and are never seen in the skin clinic. *E. floccosum* infection of the feet is comparatively uncommon in the area.

FIG. 2. *Incidence of* Microsporum canis *infection of the scalp in Bristol, 1950-60.*

The importance of tinea pedis lies not only in the frequency with which the disease itself occurs, but in the fact that an infected foot can be a focus of infection for the rest of the body. This is especially true of *T. rubrum*, where infection of the hands, nails, groin, and trunk is nearly always preceded by a simple tinea pedis. For instance, in 1958, of 20 cases of *T. rubrum* infections of the feet diagnosed, 13 (65 per cent) had associated lesions elsewhere on the body; but this was true of only 3 out of 20 cases of *T. mentagrophytes* infection (15 per cent) and 1 out of 9 cases of *E. floccosum* infection.

The epidemiology of tinea pedis in the population of Bristol has been studied in a series of surveys of schools and families, the results of which have been summed up in an article elsewhere (English, 1961). Briefly, the tinea pedis infection rate in day-school children is low (3·6 per cent) and infection is spread primarily through the local swimming bath, children who swim several times weekly being much more prone to infection than those doing so less often. National Service and sports clubs provide a further opportunity for the spread of the disease, and about one third of infected men who marry may be expected to pass the infection on to their children. In the social classes in which the son attends boarding-school, the schools themselves are more important foci of infection than the baths, owing to the opportunities of cross-infection offered by communal life. It is also likely that there is a greater spread within the family in these classes, because of the frequency with which baths are taken and the consequently increased chances of cross-infection. At present, the most frequent sources of *T. rubrum* infection appear to be family contacts and residence abroad, spread through swimming baths and schools being of less importance.

### TINEA UNGUIUM

Toe-nail infections are usually caused by *T. rubrum* (10 out of 18 cases in 1958); finger-nail infections almost always so (all cases in 1958). *T. mentagrophytes* and *E. floccosum* were occasionally found in the toe-nails. Relatively to its high total incidence on the feet, however, *T. mentagrophytes* rarely attacks the nails. The skin of he hands or feet is almost always the primary site of infection, the fungus spreading rom there to the nails. Infection of the feet usually precedes that of the hands.

### TINEA CRURIS

Of the three fungi affecting the groin, *T. rubrum* is the commonest, accounting for 1 of the 56 cases; 18 infections were due to *E. floccosum* and 7 to *T. mentagrophytes* *nterdigitale* type). Despite the predominance of *T. rubrum*, Table III shows that, elative to its total incidence, *E. floccosum* (the traditional fungus of "dhobie itch") as a special predilection for the groin. Infection of this site by *T. rubrum* is usually econdary to a foot infection but the groin is often the first and only site affected by *. floccosum*.

TABLE III

Association of fungus species with groin infection

| *T. rubrum* | | *T. mentagrophytes* (*interdigitale*) | | *E. floccosum* | |
|---|---|---|---|---|---|
| Total cases | No. of groin infections | Total cases | No. of groin infections | Total cases | No. of groin infections |
| 190 | 31 (16%) | 123 | 7 (6%) | 51 | 18 (35%) |
| Total groin infections | Primary groin infections | Total groin infections | Primary groin infections | Total groin infections | Primary groin infections |
| 31 | 10 (32%) | 7 | 3 (43%) | 18 | 13 (72%) |

Only 4 cases of tinea cruris in women were seen, 3 due to *T. rubrum* and one to *. floccosum*. In each case the feet were the primary site of infection (English and a Touche, 1957).

### RINGWORM OF THE LIMBS, TRUNK AND FACE

In this country, with its high standard of living, primary lesions of these sites due fungi of human origin are comparatively rare. In the present study, 134 of the 8 cases (65 per cent) were attributable to zoophilic fungi, and are discussed below. the anthropophilic fungi found, *Malassezia furfur*, the cause of pityriasis versi-lor, was the commnest, accounting for 40 (20 per cent) cases. Most body lesions due other fungi were secondary to infections of the feet and hands.

### RINGWORM OF ANIMAL ORIGIN

Three fungi accounted for all but one of the 276 infections of animal origin. *. canis*, the cause of cat and dog ringworm, has already been discussed in relation scalp infections; lesions of the face, limbs, trunk, and hands were also of fairly quent occurrence in both adults and children.

*T. verrucosum*, the agent of cattle ringworm, was the most commonly isolated philic fungus (143 cases, 52 per cent), despite the fact that scrapings from only a all proportion of the cases encountered were submitted from sources other than the stol General Hospital. When it is realized that very many *T. verrucosum* infections treated by the family doctor and never reach the skin clinics, the high incidence of fungus is striking. There is nearly always a history of contact with infected tle, or with buildings or other structures which cattle have touched (Main, 1959; lker, 1955).

The third zoophilic fungus to occur in a significant number of patients was *T. mentagrophytes*, which accounted for 40 of the infections in this group. Usually the only animals with which patients can trace direct contact are domestic species, on which the fungus is known to occur only rarely. But there has nearly always been contact with animal feeding stuffs, seeds, etc., or with the sacks in which these have been stored, and it seems probable that wild rodents are the normal vectors of this fungus. Nine isolates, mostly from Cornwall, were of an unusual yellow-pigmented strain, which is associated with hedgehogs (Marples, 1960).

### MONILIASIS

Species of the yeast *Candida*, especially *C. albicans*, are normal commensals of the human mucosae and digestive tract. Only when the resistance of the host is in some way lowered can these organisms reach pathogenically significant numbers. For this reason when *Candida* species are isolated in the laboratory it is often difficult to decide whether or not they are of pathogenic importance in any particular case. In Table IV, only those cases are recorded in which the fungi occurred in sufficient quantity to be found easily on microscopic examination; when microscopy was negative and only occasional colonies were isolated in culture the latter were ignored.

TABLE IV

Sites from which *Candida albicans* and other *Candida* species were isolated in 189 cases of moniliasis

| Sources: | Bristol General Hospital | | West Cornwall | | Other sources | | Total | |
|---|---|---|---|---|---|---|---|---|
| Site | *C. albicans* | Other Candidas | *C. albicans* | Other Candidas | *C. albicans* | Other Candidas | *C. albicans* | Other Candidas |
| Mouth .. | 16 | 0 | 0 | 0 | 4 | 0 | 22 | 0 |
| Lips .. .. | 7 | 1 | 1 | 0 | 1 | 0 | 9 | 1 |
| Sub-mammary | 2 | 0 | 0 | 0 | 1 | 0 | 3 | 0 |
| Feet .. .. | 21 | 2 | 4 | 0 | 5 | 3 | 28 | 4 |
| Toe-nails .. | 2 | 3 | 0 | 0 | 0 | 3 | 2 | 5 |
| Hands .. | 17 | 3 | 7 | 1 | 3 | 2 | 26 | 5 |
| Finger-nails .. | 18 | 11 | 3 | 5 | 11 | 6 | 28 | 21 |
| Ano-genital .. (adults) | 43 | 2 | 4 | 0 | 20 | 0 | 60 | 2 |
| Napkin area .. | 3 | 0 | 1 | 0 | 4 | 0 | 7 | 0 |
| Total .. | 129 | 22 | 22 | 6 | 49 | 14 | 185 | 38 |

*C. albicans* is recognized as a potential pathogen, and Table IV shows that the great majority of monilial infections are attributable to this species. In only 14 (9 per cent) out of 164 skin lesions were other species present in sufficient quantity to be considered active pathogens. But in 49 finger-nail infections, species other than *C. albicans* were isolated in 21 (43 per cent). Finger-nail-plate infections are usually secondary to chronic paronychia of fungal or bacterial origin (Whittle *et al.*, 1959), and it seems likely that most of these miscellaneous species of yeasts are secondary invaders of an already damaged nail-plate.

The number of apparently primary infections of the feet is perhaps surprising, nevertheless repeated attempts to isolate dermatophytes from these cases were unsuccessful.

It seems that ano-genital infections with *Candida* are those most frequently seen by dermatologists. Diabetes is known to predispose to infection at this site, but there is evidence in this series that an appreciable number of the infections were precipitated by the local use of antibiotics for other conditions.

### UNIDENTIFIED FUNGI

It is impossible to identify a dermatophyte species without growing it in culture. In this series 8·3 per cent of fungi failed to grow though they were visible microscopically. It is important to known the reasons for these failures, as they may often be averted by appropriate methods of taking the scrapings.

Six per cent of all specimens from which cultures failed were so small that, after microscopy, no material was left for culture. In 31 per cent the lesion had been treated with a fungicide shortly before the scrapings were taken. Of the remaining 63 per cent, nearly half were nail clippings which were not accompanied by scrapings from associated skin lesions. There appears to be some special difficulty in culturing from nails (Strauss and Kligman, 1957), and the fungus could probably have been identified in most of these cases if skin scrapings had been included. In only 33 per cent of specimens from which cultures failed (2·2 per cent of the whole series) was there no apparent explanation for the failure.

### TREATMENT

It has been pointed out that during the last few years there have been advances in the treatment of fungous infections which stress the importance of proper identification of the fungi. Some applications of this new knowledge will now be considered.

#### *Local Applications*

Several new fungicides for local application have been introduced by commercial firms, but in spite of the improved fungicidal action claimed, they have not represented any substantial advance in treatment. The most commonly prescribed fungicidal application remains Ung. acid benzoic Co. (N.F.) (modified Whitfield's ointment). Any condition underlying the fungal infection must be considered and if the eruption is eczematized, irritant fungicides should be avoided. In cases of moniliasis, treatment of a primary condition such as secondarily infected eczeme or napkin eruptions can lead to the clearing of the monilial infection. The vehicle for the fungicide is important, and moist interdigital toe spaces may call for the use of a powder, whereas dry cracking areas will do better with an ointment base. In cattle ringworm (*T. verrucosum*) in which there is usually an inflammatory response (kerion) starch poultices or frequently applied normal saline soaks are often used to clean the site and promote drainage from the infected area.

#### *Systemic Fungicides*

The introduction of the oral antifungal antibiotic griseofulvin ("Grisovin" (Glaxo) and "Fulcin" (I.C.I.)) has completely altered the treatment and prognosis in many fungous infections. Griseofulvin produces changes in the keratin which allows it to resist the growth of fungus. As new skin or nails are grown the fungus is shed in the old keratin and will not spread to that affected by griseofulvin. In ringworm of the scalp traditional treatments such as X-ray epilation are no longer necessary. All the fungi infecting the scalp, including *M. audouini*, *M. canis*, *T. scheonleini* (favus), *T. violaceum* and *T. tonsurans*, are sensitive to griseofulvin and usually clear in a few weeks or months. Once treatment is started it is as well to keep the affected hair cut as closely as possible and it is very useful to be able to follow progress of the fluorescent types by regular examinations of the scalp under the Wood's lamp. The course of cattle ringworm (*T. verrucosum*) involving the scalp, beard, and other areas is shortened by giving griseofulvin.

Formerly there was no adequate treatment for ringworm of the nails. Griseofulvin will clear finger nails after a prolonged course of up to 6–9 months' continual treatment, and obviously before starting such a course it is essential to verify the diagnosis by mycological examination. Only 25 per cent of infected toe nails are cured by griseofulvin and toe nails readily become reinfected; its value in overall management of this condition is uncertain.

Griseofulvin is of little value in the treatment of ringworm of the toe-webs, for after long periods the presence of the fungus can still be demonstrated. However, ringworm of the groin and smooth skin is readily cleared, including widespread infections due to *T. rubrum.*

The average dose of griseofulvin for adults is 250 mg. q.i.d., but when prolonged courses are necessary it is possible that smaller doses may be effective. The toxic effects include occasional headache, gastric upset, dizziness, and rashes, and very rarely a reduction in the peripheral leucocyte count has been noted but no agranulocytosis has been described.

Griseofulvin has no effect on *Candida* infections (moniliasis), and Nystatin used systemically is not absorbed sufficiently to affect the yeast on the skin. Nystatin is, however, of value as a topical preparation in some conditions in which *Candida* plays a part such as in chronic paronychia. Nystatin has no effect on the ringworm fungi.

### APPENDIX

### (a) *Specimens from Skin Lesions*

*The taking and despatch of specimens.* Fungi in skin remain viable for considerable periods provided the specimen is kept dry; there is therefore no difficulty in sending scrapings by post for diagnosis. Except in a few types of moniliasis, *swabs are useless for the diagnosis of fungous infections of the skin. Scrapings from skin lesions, clippings from nails, and the root ends of hairs must be supplied.* Satisfactory specimens can be obtained if the suggestions below are followed:

(1) No fungicide should have been applied to the lesion for at least 4 days prior to taking the scraping, preferably longer.

(2) A large quantity of material should be obtained, representative of different parts of a large lesion, and of several lesions if there are more than one.

(3) If a hairy area is involved, send skin fragments *and* individual hairs if possible.

(4) If a nail is involved, send, in a separate packet, scrapings from any associated skin lesions.

(5) Wrap specimens securely in pieces of clean paper, preferably dark coloured. This is much more satisfactory than placing them between glass slides or in test-tubes or bottles, and postage is also easier.

### (b) *Sputum, Stools, Histological Specimens*

These should, if possible, be examined on the day of collection, or failing this they should be stored in a refrigerator until examined. Specimens received by post are less satisfactory. It is not often possible to identify a fungus from a histological section alone; cultures are usually necessary. Histological specimens should not, therefore, be placed in fixative until attempts to culture a suspected fungus have been made, or alternatively a part of the specimen may be kept unfixed and sent separately for mycological examination.

I am greatly indebted to Dr. R. P. Warin and Dr. C. D. Evans for contributing the section on "Treatment" and for critical reading of the manuscript, and to Dr. Mary Dixon for her comments.

### REFERENCES

English, M. P. (1961). *Brit. med. J.*, *i*, 1086.
English, M. P., and Gibson, Mary D. (1959). Ibid., *i*, 1442.
English, M. P., and La Touche, C. J. (1957). *Brit. J. Derm.*, **69**, 311.
La Touche, C. J. (1955). *Vet. Rec.*, **67**, 666.
Main, P. T. (1959). *Practitioner*, **182**, 347.
Marples, M. J. (1959). *New Zealand med. J.*, **58**, 64.
Marples, M. J., and Smith, J. M. B. (1960). *Nature*, **188**, 867.
Strauss, J. S., and Kligman, A. M. (1957). *Arch. Derm., Chicago*, **76**, 70.
Walker, J. (1955). *Brit. med. J. ii*, 1430.
Whittle, C. H., Moffatt, J. L. and Davis, R. A. (1959). *Brit. J. Derm.*, **71**, 1.

## Figures and Tables

**Fig. 1. f1:**
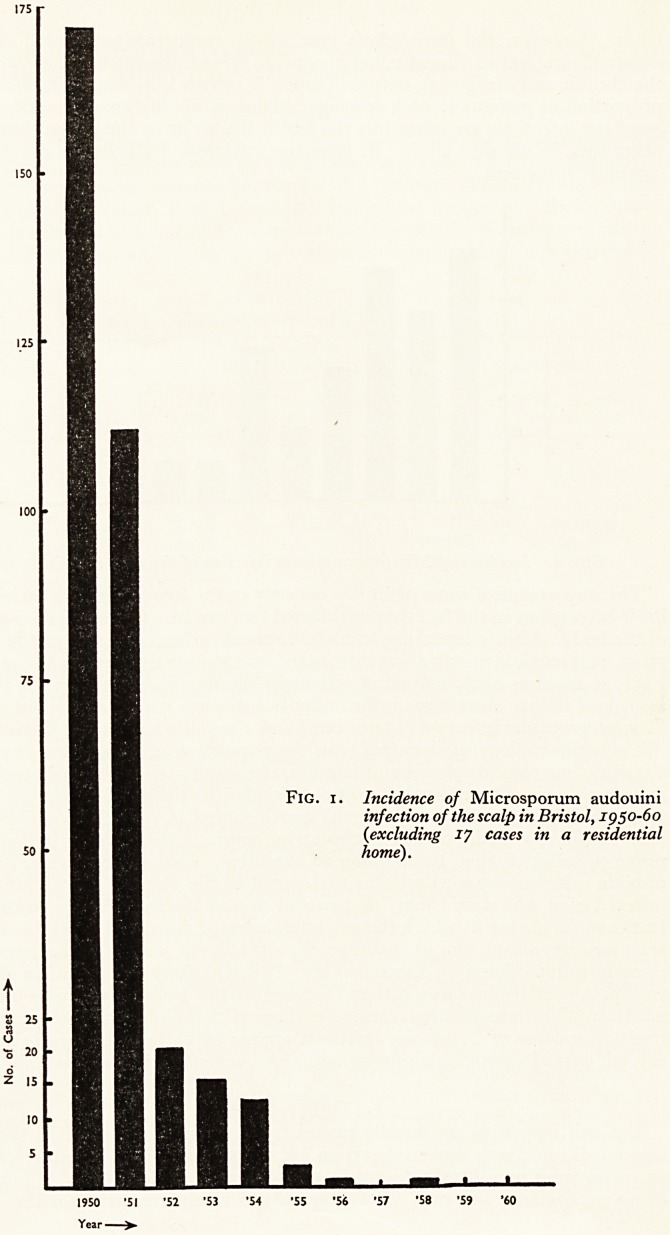


**Fig. 2. f2:**